# Clinical severity predominates over routine inflammatory and metabolic biomarkers in predicting hemorrhagic transformation after intravenous thrombolysis: internal temporal validation of incremental biomarker value

**DOI:** 10.3389/fneur.2026.1940110

**Published:** 2026-10-21

**Authors:** Huan Liang, Baiyu Zeng, Jingquan Wang, Lizhen Yi, Linxing Huang, Ziyan Lin, Cai Zhou, Zenghui Yue

**Affiliations:** 1 College of Acupuncture-Moxibustion, Tuina and Rehabilitation, Hunan University of Chinese Medicine, Changsha, China; 2 Teaching and Research Office of TCM Internal Medicine, Hunan Traditional Chinese Medical College, Zhuzhou, China; 3 Changsha Central Hospital, Changsha, Hunan, China; 4 School of Physical Education, Hunan University of Arts and Science, Changde, China

**Keywords:** acute ischemic stroke, clinical severity, hemorrhagic transformation, incremental predictive value, internal temporal validation, intravenous thrombolysis, neutrophil-to-lymphocyte ratio

## Abstract

**Background and Objective:**

Hemorrhagic transformation (HT) remains an important safety concern after intravenous thrombolysis for acute ischemic stroke. We evaluated whether routine inflammatory and metabolic biomarkers improve HT prediction beyond clinical features.

**Methods:**

We analyzed 847 thrombolysis-treated admissions from 2020 to 2026. In-hospital imaging-confirmed HT was the primary outcome, and strict 7-day ECASS II-compatible symptomatic intracranial hemorrhage (sICH) was exploratory. Admission date defined derivation (*n* = 593; 67 HT events) and internal temporal-validation (*n* = 254; 27 HT events) cohorts. The six-variable clinical model included age, baseline NIHSS, atrial fibrillation, large-vessel occlusion, the rapid-glucose field, and onset-to-treatment time. Models added ln(NLR), AIP plus residual cholesterol, or all three biomarkers. Twenty imputed datasets were generated using derivation-fitted imputation models. We assessed discrimination, calibration, Brier score, decision curves, and sensitivity analyses.

**Results:**

HT occurred during 94 of 847 admissions (11.1%). The clinical model yielded AUCs of 0.812 (95% CI 0.761–0.860) in derivation and 0.801 (0.707–0.881) in temporal validation. Adding ln(NLR) increased the validation AUC to 0.818 (ΔAUC +0.017; DeLong *p* = 0.220). Adding AIP and residual cholesterol yielded an AUC of 0.803 (ΔAUC +0.002; *p* = 0.818). The full model yielded AUCs of 0.817 in derivation and 0.818 in validation (ΔAUC +0.018; *p* = 0.346). Baseline NIHSS was independently associated with HT (OR 1.084 per point, 95% CI 1.044–1.126; *p* < 0.001). Atrial fibrillation was independently associated with HT (OR 2.66, 95% CI 1.34–5.27; *p* = 0.005). The biomarkers ln(NLR), AIP, and residual cholesterol were not independently associated with HT. Validation calibration was similar, with a Brier score of 0.081 for both models. Biomarker gains were inconsistent across sensitivity analyses. In the exploratory sICH analysis (37 events), ln(NLR) was associated with sICH (OR 1.89, 1.04–3.42; *p* = 0.037). Validation included only 12 events, and the AUC increment was not statistically significant (*p* = 0.097).

**Conclusion:**

Clinical severity and established clinical features provided most of the predictive information for post-thrombolysis HT. Routine ln(NLR), AIP, and residual cholesterol did not provide sufficiently robust incremental predictive value beyond the clinical model. The NLR–sICH signal remains exploratory and requires external validation.

## 1 Introduction

Stroke remains a major global cause of death and disability, with ischemic stroke accounting for most incident cases (1). For eligible patients with acute ischemic stroke, rapid reperfusion is central to preserving threatened brain tissue. Intravenous thrombolysis with alteplase or tenecteplase remains a mainstay of acute treatment, and benefit is strongly time dependent (2, 3). Its most feared complication is hemorrhagic transformation, particularly symptomatic intracranial hemorrhage, which is strongly associated with death and disability (4).

Predicting clinically important bleeding before it becomes apparent remains difficult. Age, baseline NIHSS, glucose, and early imaging signs are incorporated into established bleeding-risk scores such as HAT and SEDAN (5, 6). However, multicenter evaluations have shown that existing scores generally provide only modest discrimination (7). This leaves uncertainty about whether routinely available biomarkers can add useful information beyond clinical severity.

Inflammation and endothelial injury provide a plausible biological link between ischemia–reperfusion and hemorrhagic transformation (8, 9). Higher NLR has been associated with post-thrombolysis hemorrhagic transformation, but results vary with sampling time and outcome definition (10). Lipid studies have reported inverse associations between cholesterol measures and hemorrhagic transformation after recanalization therapy and in selected stroke subtypes (11, 12). More recent cohorts identified similar signals for AIP without reperfusion and for remnant cholesterol after intravenous thrombolysis (13, 14). These findings establish biological and prognostic associations, but they do not show whether biomarkers improve a clinical prediction model.

The incremental question is clinically important because the strength of an association does not by itself establish useful patient-level classification. A biomarker can be associated with outcome yet add little discrimination, calibration, or decision value (15, 16). Temporal validation is also relevant when laboratory availability and treatment practice change over time (15). We therefore evaluated routine ln(NLR), AIP, and residual cholesterol within models anchored to age, NIHSS, AF, LVO, glucose, and treatment delay. We compared discrimination, calibration, decision-curve net benefit, and sensitivity analyses across a strict chronological split.

## 2 Materials and methods

### 2.1 Study design and population

This single-center retrospective cohort study included adults with acute ischemic stroke treated with intravenous thrombolysis at Changsha Central Hospital between January 2020 and May 2026. The analytical extract contained 851 records. Reconciliation of hospitalization records identified four duplicate entries, leaving 847 unique admissions, all with an admission date. Intravenous thrombolysis during the study period was performed with alteplase or tenecteplase; no other thrombolytic agents were used. Tenecteplase is an established alternative to alteplase in eligible patients (17). In some archived records, treatment was documented only as “intravenous thrombolysis” without naming the agent, so individual agent identity could not be reconstructed reliably for every admission and was not included in the primary prediction models. The Institutional Review Board of Changsha Central Hospital approved the study (KY-2026-219-01) and waived informed consent because the data were retrospective and de-identified.

### 2.2 Outcome definitions

The primary outcome was in-hospital imaging-confirmed HT after thrombolysis. HT was the umbrella imaging outcome under the ECASS framework (18). Hemorrhagic infarction (HI) and parenchymal hematoma (PH) were radiological subtypes, whereas sICH was a distinct clinical-radiological endpoint; this separation is consistent with contemporary bleeding classification after reperfusion therapy (19). Routine imaging reports classified all 94 HT events as either HI (*n* = 56) or PH (*n* = 38). Detailed HI1/HI2 or PH1/PH2 subclassification was accepted only when supported by specific report wording. This level of detail was available for 26 events and was not analyzed further.

The exploratory secondary endpoint was sICH, defined according to ECASS II as intracranial hemorrhage within 7 days accompanied by an NIHSS increase of at least four points or death (20). After source review, all hemorrhage-positive admissions had sufficient neurological information to determine whether the ECASS II clinical criterion (NIHSS increase ≥4 points or death) was met. Patients without intracranial hemorrhage were classified as not meeting the sICH definition, yielding 37 events (25 in derivation and 12 in temporal validation). Hemorrhages first documented after 7 days were excluded from this strict endpoint.

HT was abstracted from official hospital radiology reports, without author rereading. Targeted source-record re-abstraction was used to resolve ambiguous documentation and recover incomplete sampling-time information and was not formally blinded to HT status. HT classification remained based on the official radiology reports rather than author reinterpretation of imaging. Archived images were not uniformly retrievable for blinded dual review; central adjudication and inter-rater agreement were unavailable. Imaging intervals varied, and repeat scans could be routine or clinically triggered. Archived documentation supported 91 of 94 events; excluding the remaining three yielded similar results. A 7-day HT sensitivity analysis addressed 21 events first documented after day 7 and three with missing timing.

### 2.3 Candidate predictors

The primary analysis used a clinically anchored nested-model framework. The clinical model included age, baseline NIHSS, atrial fibrillation (AF), large-vessel occlusion (LVO), the source-database rapid-glucose field, and onset-to-treatment time (OTT). Baseline neurological severity was quantified with the NIHSS (21). These variables capture established clinical risk information; prior evidence particularly supports stroke severity and glucose-related risk after thrombolysis (22, 23). HAT and SEDAN were not formally evaluated because several required early-imaging components could not be reconstructed uniformly across the cohort. Rapid blood glucose was obtained before IVT as part of the routine emergency assessment. Fasting glucose was reported descriptively in Table 1 but excluded from prediction models because it represented a separate, less uniformly timed measurement.

**Table 1:** Baseline characteristics stratified by hemorrhagic transformation.

Variable	All (*n* = 847)	HT+ (*n* = 94)	HT− (*n* = 753)	*p*-value
Demographics and clinical				
Age, years	67.50 [58.00–75.25]	70.00 [62.00–80.00]	67.00 [58.00–75.00]	0.022
Male	538 (64.9)	55 (59.1)	483 (65.6)	0.217
Baseline NIHSS	6.00 [3.00–12.00]	15.00 [10.00–20.00]	5.00 [3.00–11.00]	<0.001
Onset-to-treatment, h	4.00 [3.00–6.00]	4.00 [3.00–5.00]	4.00 [3.00–6.00]	0.189
Large-vessel occlusion	329 (45.4)	63 (75.9)	266 (41.4)	<0.001
Atrial fibrillation	161 (19.1)	43 (46.2)	118 (15.7)	<0.001
Hypertension	665 (78.9)	72 (77.4)	593 (79.1)	0.714
Diabetes	262 (31.1)	37 (39.4)	225 (30.1)	0.067
Prior stroke/TIA	144 (17.2)	17 (18.3)	127 (17.1)	0.771
Bridging thrombectomy	76 (9.0)	25 (26.6)	51 (6.8)	<0.001
Rapid blood glucose, mmol/L	6.90 [6.00–9.07]	6.80 [6.10–9.25]	6.90 [6.00–9.00]	0.981
Fasting blood glucose, mmol/L	6.09 [5.03–7.76]	7.16 [5.74–9.96]	5.93 [4.93–7.47]	<0.001
Inflammatory markers				
SII, ×10^9^/L	563.00 [346.00–885.50]	644.50 [409.25–1114.25]	548.00 [342.00–862.00]	0.030
SIRI	1.08 [0.69–1.81]	1.31 [0.87–2.40]	1.04 [0.67–1.71]	0.004
NLR	2.67 [1.82–4.05]	3.30 [2.25–5.49]	2.60 [1.77–3.94]	<0.001
Metabolic markers				
TyG index	8.88 [8.45–9.40]	8.85 [8.43–9.38]	8.89 [8.45–9.40]	0.829
AIP	0.12 [−0.05–0.31]	0.08 [−0.17–0.19]	0.12 [−0.03–0.32]	0.015
Residual cholesterol, mmol/L	0.52 [0.23–0.83]	0.50 [0.19–0.70]	0.53 [0.24–0.84]	0.144
LDL-C, mmol/L	2.71 [2.15–3.24]	2.43 [1.73–2.97]	2.73 [2.20–3.26]	<0.001
Total cholesterol, mmol/L	4.40 [3.70–5.12]	4.10 [3.24–4.73]	4.43 [3.73–5.15]	0.002
Triglycerides, mmol/L	1.46 [1.01–2.02]	1.20 [0.85–1.70]	1.48 [1.04–2.04]	0.005
HDL-C, mmol/L	1.07 [0.90–1.27]	1.19 [0.95–1.31]	1.06 [0.89–1.27]	0.030

Continuous variables are median [interquartile range] and were compared with the Mann–Whitney U test. Categorical variables are *n* (%) and were compared with the *χ*^2^ or Fisher exact test. HT, hemorrhagic transformation; NIHSS, National Institutes of Health Stroke Scale; SII, systemic immune-inflammation index; SIRI, systemic inflammation response index; NLR, neutrophil-to-lymphocyte ratio; AIP, atherogenic index of plasma; LDL-C/HDL-C, low−/high-density lipoprotein cholesterol. Categorical percentages use observed cases, so denominators vary by variable. The rapid-glucose field represents the measurement obtained before IVT during routine emergency assessment. Fasting glucose is shown descriptively (681/847 observed) because it was a separate, less uniformly timed measurement and was not included in the prediction models.

Three biomarkers were evaluated for incremental value: ln(NLR), AIP, and residual cholesterol. NLR was calculated as the absolute neutrophil count divided by the absolute lymphocyte count (24, 25). SII was calculated as neutrophil count × platelet count / lymphocyte count, and SIRI as neutrophil count × monocyte count / lymphocyte count. AIP was defined as log10(triglycerides/HDL-C) using molar concentrations (26). The TyG index, used descriptively only, was calculated as ln[fasting triglycerides (mg/dL) × fasting glucose (mg/dL)/2]. Residual cholesterol was calculated as total cholesterol minus directly measured LDL-C minus HDL-C. LDL-C was not calculated with the Friedewald equation, and its measurement method was unchanged during the study. The laboratory analyzers used for the hematological and lipid measurements remained unchanged across the July 2024 EMR migration. SII, SIRI, TyG, and the conventional lipid profile were retained for descriptive analyses but were not forced into the primary model.

According to institutional thrombolysis workflow and the source-field definitions, CBC- and lipid-derived variables were calculated from the initial emergency blood-sampling episode, which occurred before IVT; subsequent inpatient repeat measurements were not included. Exact draw-to-bolus intervals were not retained for every admission, and laboratory results, particularly lipid results, were not necessarily available before treatment. These variables were therefore considered to reflect pre-IVT specimens but were not assumed to be universally available for real-time pre-bolus decision-making. The conservative timing sensitivity analysis was retained as an additional robustness check by treating biomarker values with incomplete archived sampling documentation as missing. Bridging thrombectomy was addressed separately in an exclusion sensitivity analysis.

### 2.4 Handling of missing data

Missing data were handled using multiple imputation. This approach can reduce bias and preserve uncertainty when its assumptions are plausible (27). Twenty iterative posterior-sampling imputations were fitted using derivation-cohort predictors only, then applied to temporal validation to limit information leakage. Imputed AF and LVO values were dichotomized at 0.5. Continuous variables were winsorized at the derivation-cohort 1st and 99th percentiles, and the same bounds were applied to validation. Regression coefficients and standard errors were pooled using Rubin-style within- and between-imputation variance. Performance metrics were calculated from the mean predicted probability across imputations. Table 2 summarizes missingness before imputation.

**Table 2:** Missing-data proportions and temporal distribution of predictors in the primary models.

Predictor	Missing, all (%)	Missing, deriv. (%)	Missing, valid. (%)	Derivation	Temporal validation
Age	0.4	0.3	0.4	68 [58–76]	67 [58–74]
Baseline NIHSS	2.6	0.5	7.5	6 [3–13]	5 [3–11]
Atrial fibrillation	0.2	0.3	0.0	123/591 (20.8%)	38/254 (15.0%)
Large-vessel occlusion	14.4	14.8	13.4	221/505 (43.8%)	108/220 (49.1%)
Rapid glucose	16.6	13.0	25.2	6.9 [6.0–8.8]	6.8 [6.0–9.4]
Onset-to-treatment time	15.0	7.4	32.7	4.42 [3–6]	4.0 [3–5]
NLR	16.8	9.9	32.7	2.62 [1.76–3.77]	3.05 [1.90–5.86]
AIP	11.6	8.6	18.5	0.12 [−0.07–0.31]	0.13 [0.00–0.27]
Residual cholesterol	12.0	8.8	19.7	0.39 [0.17–0.71]	0.78 [0.57–1.08]

Continuous variables are median [IQR], and binary variables are *n* (%) among observed cases. Missingness is reported before multiple imputation. NIHSS, rapid glucose, OTT, and NLR had more missing values in temporal validation, which also showed a marked shift in residual cholesterol. AF, atrial fibrillation; LVO, large-vessel occlusion; NLR, neutrophil-to-lymphocyte ratio; AIP, atherogenic index of plasma; RC, residual cholesterol.

### 2.5 Temporal split and statistical analysis

After duplicate removal, admissions were ordered by admission date, and cohort assignment was generated from chronological rank. The earliest 70% formed the derivation cohort (*n* = 593; 67 HT events; January 2020 to 25 November 2024). The latest 30% formed the internal temporal-validation cohort (*n* = 254; 27 HT events; 28 November 2024 to May 2026). The boundary fell between chronological ranks 593 and 594, corresponding to admissions on 25 and 28 November 2024, respectively. No undated record was assigned to either cohort. The institutional electronic medical record system was migrated to a new platform in July 2024. This single-center internal temporal validation assesses robustness to within-institution temporal drift but not geographic transportability (15, 28). Source-date reconciliation and the descriptive comparison of records with and without admission dates in the original extract are summarized in Supplementary Tables S2, S3. Continuous variables were compared with the Mann–Whitney U test and reported as median [IQR]. Categorical variables were compared with the *χ*^2^ or Fisher exact test and reported as *n* (%).

Four logistic models were evaluated. The clinical model included age, baseline NIHSS, AF, LVO, rapid glucose, and OTT. The inflammation-augmented model added ln(NLR), and the metabolic-augmented model added AIP and residual cholesterol. The full model included all nine variables. This design tested incremental biomarker value directly, without LASSO selection of a final biomarker score. Adjusted associations were estimated from the full model. Dependence between AIP and residual cholesterol was assessed using Pearson and Spearman correlations and variance-inflation factors. Correlations were moderate (Pearson r = 0.475; Spearman *ρ* = 0.483), and the maximum VIF was 1.54.

Full-model specification: logit[P(HT)] = β₀ + β₁(Age) + β₂(NIHSS) + β₃(AF) + β₄(LVO) + β₅(Rapid glucose) + β₆(OTT) + β₇ ln(NLR) + β₈(AIP) + β₉(Residual cholesterol). Non-intercept coefficients are reported in Table 3; this expression is provided to make the model structure explicit, not to define a deployable clinical risk score. All continuous predictors were entered as linear terms on the logit scale, except NLR, which was natural-log transformed before modeling (Figure 1).

**Table 3:** Pooled multivariable logistic regression for hemorrhagic transformation in the full nine-variable model fitted in the derivation cohort.

Variable	*β*	OR (95% CI)	*p*-value
Age (per +1 year)	−0.007	0.99 (0.97–1.02)	0.597
Baseline NIHSS (per +1)	0.081	1.08 (1.04–1.13)	<0.001
Atrial fibrillation (yes vs. no)	0.979	2.66 (1.34–5.27)	0.005
Large-vessel occlusion (yes vs. no)	0.558	1.75 (0.88–3.45)	0.109
Rapid glucose (per +1 mmol/L)	0.000	1.00 (0.90–1.11)	0.994
Onset-to-treatment time (per +1 h)	0.037	1.04 (0.94–1.15)	0.478
ln(NLR) (per +1)	0.236	1.27 (0.83–1.93)	0.275
AIP (per +1)	−0.351	0.70 (0.20–2.43)	0.578
Residual cholesterol (per +1 mmol/L)	−0.122	0.88 (0.37–2.12)	0.784

*β*, pooled regression coefficient; OR, odds ratio; CI, confidence interval. Coefficients and standard errors were pooled across 20 imputations in the derivation cohort. ln(NLR) denotes the natural logarithm of NLR; residual cholesterol is expressed in mmol/L. Rapid glucose was obtained before IVT during routine emergency assessment.

**Figure 1:**
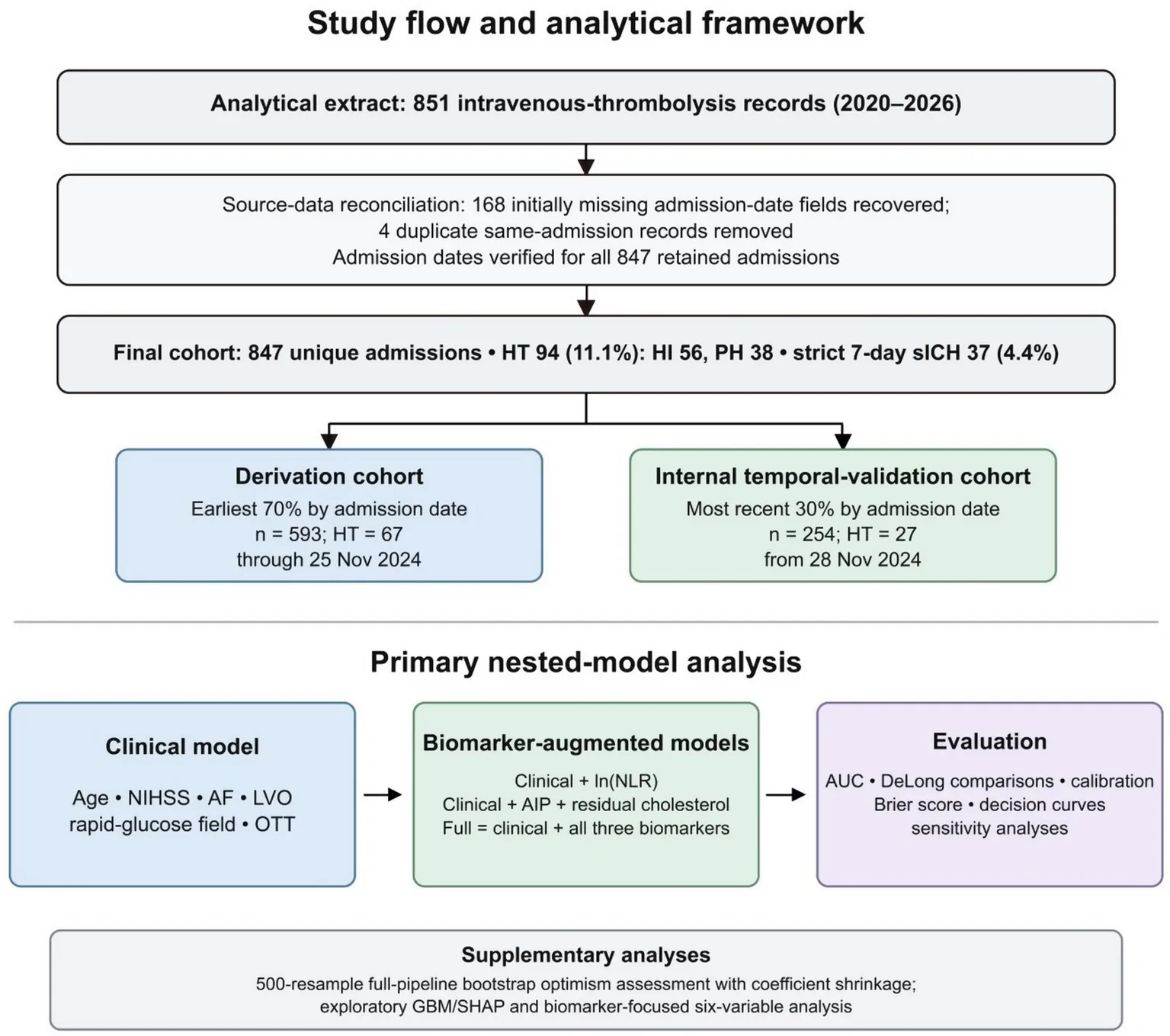
Study flow and analytical framework. The analytical extract contained 851 records. Four duplicate admissions were removed, leaving 847 unique admissions with available admission dates. The earliest 70% formed the derivation cohort (*n* = 593; 67 HT events; through 25 November 2024). The latest 30% formed the internal temporal-validation cohort (*n* = 254; 27 HT events; from 28 November 2024). Nested logistic models tested the incremental value of ln(NLR), AIP, and residual cholesterol over a six-variable clinical model. Supplementary analyses addressed internal shrinkage and exploratory model interpretation.

Model performance was assessed in both cohorts using AUC, calibration slope and intercept, observed-to-expected (O:E) ratio, and their 95% confidence intervals. Calibration measures were interpreted according to current guidance (29). We also calculated the Brier score and performed decision-curve analysis (30). Correlated AUCs were compared with DeLong’s method (31). Sensitivity analyses excluded bridging thrombectomy, restricted LVO analyses to patients with observed LVO status, and excluded three HT events lacking re-verifiable archived documentation. Additional analyses restricted HT to events within 7 days and used the documentation-conservative laboratory-timing approach described above. As an exploratory complementary interpretation, gradient boosting with SHAP values was applied in the same 847-admission cohort using six variables: baseline NIHSS, AF, LVO, ln(NLR), AIP, and residual cholesterol (32). A concise mean-|SHAP| summary is shown in Figure 2D, and the full SHAP distribution is provided in Supplementary Figure S2. This same-cohort analysis was not an independent validation and did not inform predictor selection or model-superiority claims.

**Figure 2:**
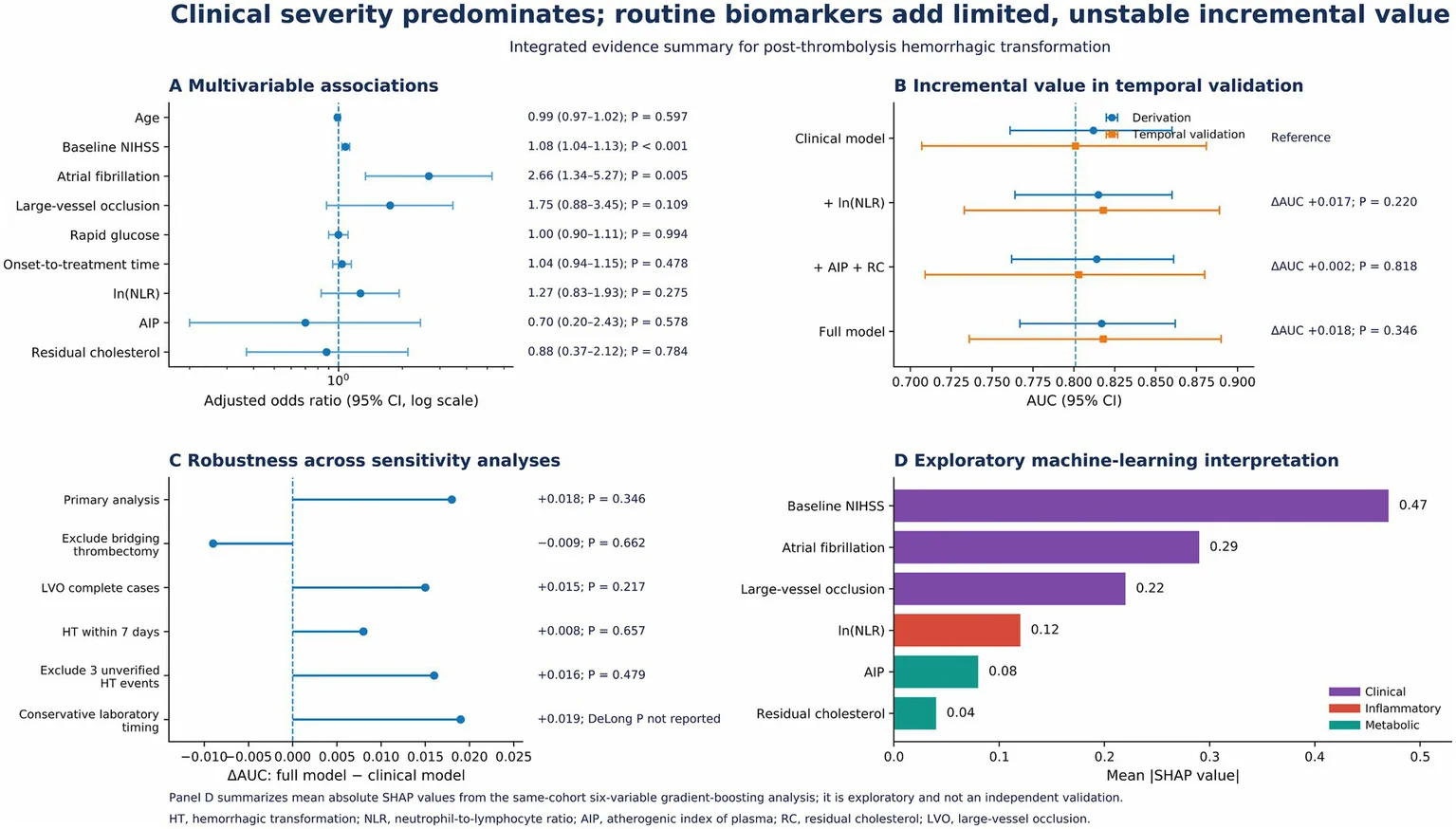
Integrated evidence summary of multivariable associations, incremental predictive value, robustness, and exploratory complementary machine-learning interpretation. **(A)** Adjusted odds ratios with 95% confidence intervals from the full nine-variable model. **(B)** AUCs with 95% confidence intervals for the clinical, inflammation-augmented, metabolic-augmented, and full models in derivation and temporal validation; displayed ΔAUC and *p* values refer to temporal-validation comparisons with the clinical model. **(C)** Change in validation AUC for the full versus clinical model across the primary and sensitivity analyses. **(D)** Mean absolute SHAP values from the same-cohort six-variable gradient-boosting analysis using baseline NIHSS, AF, LVO, ln(NLR), AIP, and residual cholesterol. Panel D is exploratory and is not an independent validation of the primary nested logistic models. NLR in the model labels denotes ln(NLR); RC, residual cholesterol.

This retrospective study included all eligible records and did not use a prospectively determined sample size. After source cleaning, the derivation cohort contained 593 admissions and 67 HT events. The full model contained nine parameters, corresponding to approximately 7.4 events per parameter. We therefore conducted a post-hoc adequacy assessment using the Riley framework for binary-outcome prediction models (33). Given an apparent Cox–Snell *R*^2^ of 0.113 and a target shrinkage factor of 0.90, the shrinkage criterion indicated approximately 671 development observations. This exceeded the 593 available admissions. Because this calculation used observed model fit, it was not a prospective sample-size determination. Nevertheless, it reinforced concerns about coefficient precision and overfitting. We therefore framed the study as an exploratory incremental-value analysis with internal temporal validation, not as development of a clinically ready tool.

We also performed supplementary bootstrap internal validation of the nine-variable full model using 500 resamples (15). Within each resample, we re-estimated the 1st and 99th percentile winsorization limits and refitted iterative chained-equation imputation in the resampled derivation data. Imputed AF and LVO values were re-dichotomized at 0.5, and the logistic model was refitted before evaluation in the full derivation sample. For each resample, performance was calculated both in the bootstrap sample and after applying the bootstrap-fitted preprocessing/model pipeline to the original derivation cohort. Optimism was defined as the difference between these two estimates and averaged across the 500 resamples. The primary nested-model analysis retained the 20 posterior-sampling imputations. The bootstrap was a supplementary overfitting check that repeated preprocessing and model fitting rather than holding a fixed imputation or model. We estimated optimism in AUC and the calibration slope across resamples. The mean calibration slope provided a uniform shrinkage factor for the nine non-intercept coefficients, reported in Supplementary Table S1.

Analyses were performed in Python 3.12 using NumPy, pandas, SciPy, scikit-learn, and custom statistical routines. All preprocessing parameters were estimated in derivation and then applied to temporal validation. Inference emphasized discrimination, calibration, Brier score, decision curves, and consistency across sensitivity analyses.

## 3 Results

### 3.1 Baseline characteristics

After deduplication, 847 unique admissions were analyzed; 94 admissions (11.1%) had in-hospital imaging-confirmed HT. HT occurred in 67 of 593 derivation admissions (11.3%) and 27 of 254 validation admissions (10.6%). Strict 7-day sICH occurred during 37 admissions, including 25 in derivation and 12 in validation. Among the 94 HT events, 56 (59.6%) were HI and 38 (40.4%) were PH. Detailed HI1/HI2 or PH1/PH2 subclassification was supported for only 26 events and was not analyzed further.

Patients with HT were older than those without HT (median 70 versus 67 years; *p* = 0.022) and had higher baseline NIHSS scores (15 versus 5; *p* < 0.001). They also more often had LVO (75.9% versus 41.4%; *p* < 0.001) and AF (46.2% versus 15.7%; *p* < 0.001). Bridging thrombectomy was also more frequent (26.6% versus 6.8%; *p* < 0.001). Rapid glucose did not differ between groups (6.80 [6.10–9.25] versus 6.90 [6.00–9.00] mmol/L; *p* = 0.981). Fasting glucose was available for 681 admissions and was reported descriptively because its timing was less standardized. It was higher in patients with HT (7.16 [5.74–9.96] versus 5.93 [4.93–7.47] mmol/L; *p* < 0.001).

SII, SIRI, and NLR were higher in patients with HT. LDL-C, total cholesterol, triglycerides, and AIP were lower, whereas HDL-C was higher. Residual cholesterol showed the same inverse direction but was not statistically significant (Table 1).

### 3.2 Nested model specification and discrimination

The six-variable clinical model showed good discrimination, with AUCs of 0.812 (95% CI 0.761–0.860) in derivation and 0.801 (0.707–0.881) in temporal validation. Adding ln(NLR) increased the validation AUC to 0.818 (ΔAUC +0.017; DeLong *p* = 0.220). Adding AIP and residual cholesterol without NLR yielded a validation AUC of 0.803 (ΔAUC +0.002; *p* = 0.818). The full model yielded AUCs of 0.817 (0.767–0.862) in derivation and 0.818 (0.736–0.890) in validation. Its validation ΔAUC was +0.018 versus the clinical model (*p* = 0.346). Thus, biomarkers produced small numerical changes without statistically robust incremental discrimination (Figure 3; Table 4).

**Figure 3:**
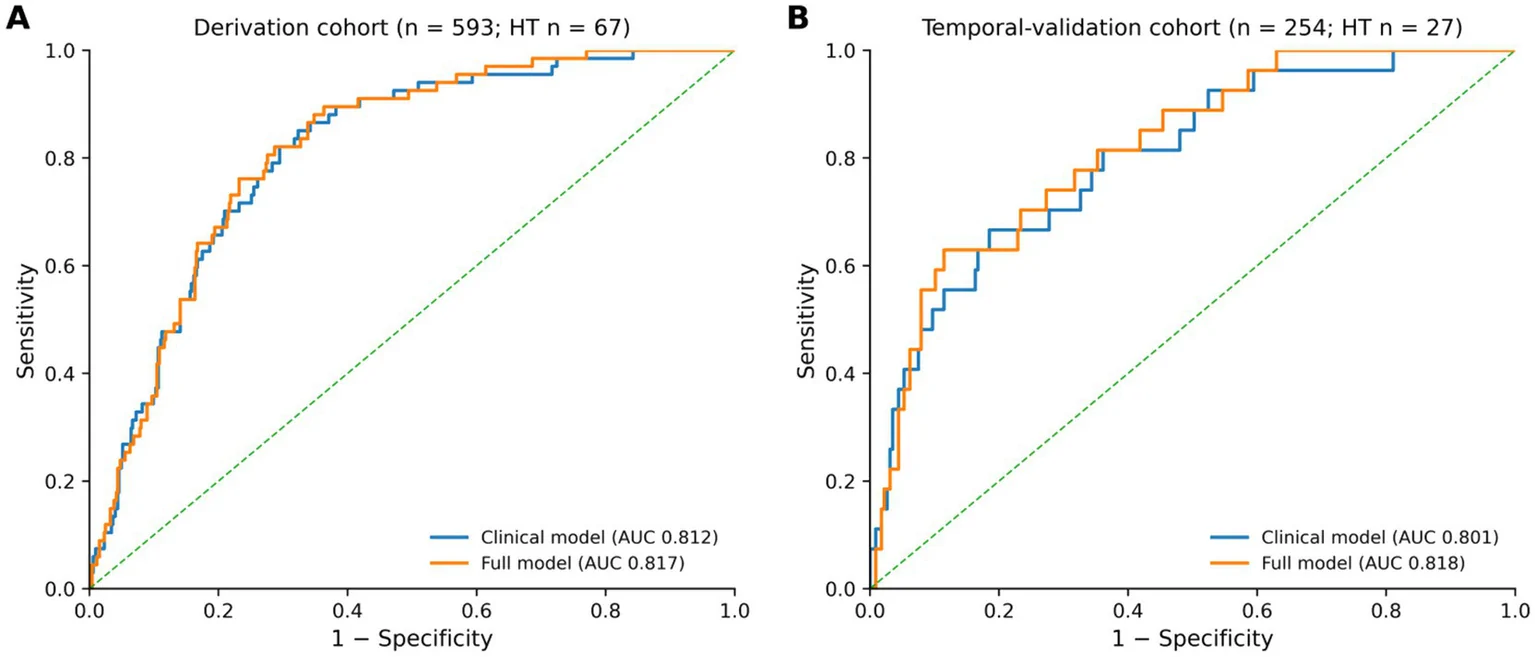
Receiver operating characteristic curves for the nested-model analysis. **(A)** Derivation cohort (*n* = 593; 67 HT events). **(B)** Internal temporal-validation cohort (*n* = 254; 27 HT events). The clinical model included age, baseline NIHSS, AF, LVO, rapid glucose, and OTT. The full model additionally included ln(NLR), AIP, and residual cholesterol.

**Table 4:** Discrimination and calibration of the nested models.

Metric	Derivation	Temporal validation
Discrimination (AUC, 95% CI)		
Clinical model	0.812 (0.761–0.860)	0.801 (0.707–0.881)
Clinical model + ln(NLR)	0.815 (0.764–0.860)	0.818 (0.733–0.889); Δ + 0.017; *p* = 0.220
Metabolic/full-model comparisons		
Clinical model + AIP + RC	0.814 (0.762–0.861)	0.803 (0.709–0.880); Δ + 0.002; *p* = 0.818
Full model	0.817 (0.767–0.862)	0.818 (0.736–0.890); Δ + 0.018; *p* = 0.346
Brier score – clinical model	—	0.081
Brier score – full model	—	0.081
Clinical model calibration		
Calibration intercept	—	0.059 (−0.376 to 0.494)
Calibration slope	—	1.029 (0.642–1.416)
Full model calibration		
Calibration intercept	—	0.053 (−0.381 to 0.488)
Calibration slope	—	1.060 (0.669–1.452)
Observed-to-expected ratio – clinical model	—	1.045 (0.689–1.521)
Observed-to-expected ratio – full model	—	1.041 (0.686–1.515)

For validation rows, ΔAUC and DeLong *p*-values compare each biomarker-augmented model with the six-variable clinical model. AUC, area under the receiver operating characteristic curve; NLR, neutrophil-to-lymphocyte ratio; AIP, atherogenic index of plasma; RC, residual cholesterol.

### 3.3 Multivariable associations

In the derivation cohort, baseline NIHSS and AF showed the clearest independent associations with HT in the full nine-variable model. Each one-point increase in NIHSS was associated with 8.4% higher odds of HT (OR 1.084, 95% CI 1.044–1.126; *p* < 0.001). AF was associated with approximately 2.7-fold higher odds (OR 2.66, 1.34–5.27; *p* = 0.005). LVO showed a positive but imprecise association (OR 1.75, 0.88–3.45; *p* = 0.109). Age, rapid glucose, and OTT were not independently associated with HT. Associations were attenuated for ln(NLR) (OR 1.27, 0.83–1.93; *p* = 0.275) and AIP (OR 0.70, 0.20–2.43; *p =* 0.578). Residual cholesterol was also attenuated (OR 0.88, 0.37–2.12; *p =* 0.784) (Table 3; Figure 2A).

### 3.4 Bootstrap internal validation and coefficient shrinkage

In the 500-resample bootstrap analysis, the apparent derivation AUC of the full model was 0.817. Mean optimism was 0.031, yielding an optimism-corrected derivation AUC of approximately 0.786. The mean calibration slope was 0.863 (median 0.855), supporting a uniform shrinkage factor of approximately 0.86. Shrinkage attenuated coefficient magnitudes but preserved the overall pattern: baseline NIHSS and AF remained the dominant clinical signals, whereas ln(NLR), AIP, and residual cholesterol remained weaker. Supplementary Table S1 presents the shrunken estimates. The temporal-validation estimate remained the primary measure of out-of-time discrimination (Table 5).

**Table 5:** Exploratory NLR-augmented model for 7-day symptomatic intracranial hemorrhage in the derivation cohort.

Variable	OR	95% CI	*p*-value
Baseline NIHSS (per +1)	1.07	1.02–1.13	0.010
Atrial fibrillation	2.35	0.98–5.63	0.055
Large-vessel occlusion	1.80	0.63–5.18	0.274
ln(NLR)	1.89	1.04–3.42	0.037

Adjusted odds ratios were estimated from the NLR-augmented model fitted in the derivation cohort. Because event counts were limited, the exploratory clinical reference model was intentionally restricted to baseline NIHSS, AF, and LVO. The fitted NLR-augmented model yielded AUCs of 0.823 in derivation and 0.888 in temporal validation. Its validation AUC increase was +0.071 (DeLong *p* = 0.097). sICH, symptomatic intracranial hemorrhage.

### 3.5 Calibration

Calibration was similar for the clinical and full models in temporal validation. The clinical model had a Brier score of 0.081 and a calibration intercept of 0.059 (95% CI − 0.376 to 0.494). Its calibration slope was 1.029 (0.642–1.416), and its O:E ratio was 1.045 (0.689–1.521). The corresponding full-model values were 0.081, 0.053 (−0.381 to 0.488), 1.060 (0.669–1.452), and 1.041 (0.686–1.515) (Figure 4 displays the calibration curves for the full model, whereas Table 4 reports numerical calibration estimates for both the clinical and full models).

**Figure 4:**
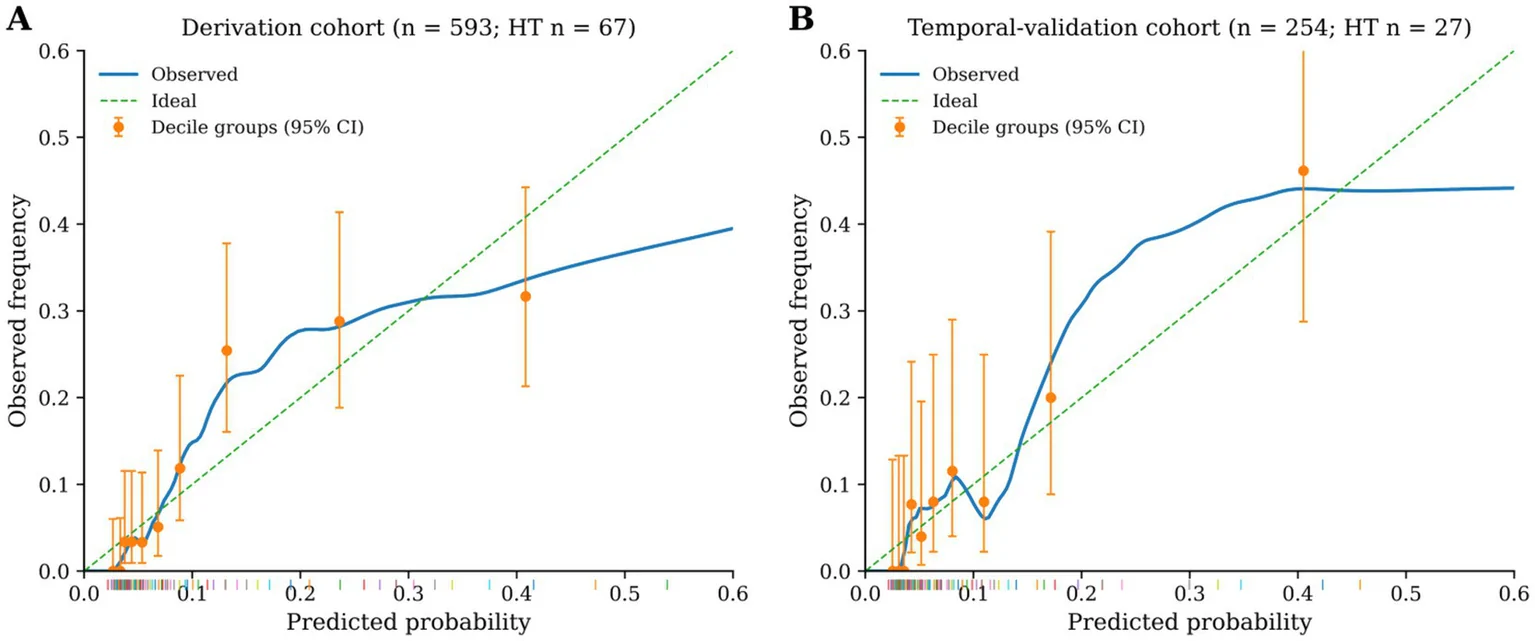
Calibration curves for the full model. **(A)** Derivation cohort. **(B)** Internal temporal-validation cohort. Solid curves show smoothed observed risk, and points show grouped observed proportions with 95% confidence intervals. The dashed diagonal indicates ideal calibration, and the bottom rug shows the distribution of predicted risk.

### 3.6 Clinical decision value

Decision-curve analysis showed closely similar net-benefit trajectories for the clinical and full models in both cohorts (Figure 5). No broad or consistently separated net-benefit advantage was observed for the full model across the examined threshold range. Supplementary Figure S1 presents decision curves for the exploratory six-variable biomarker-focused model in the 847-admission cohort.

**Figure 5:**
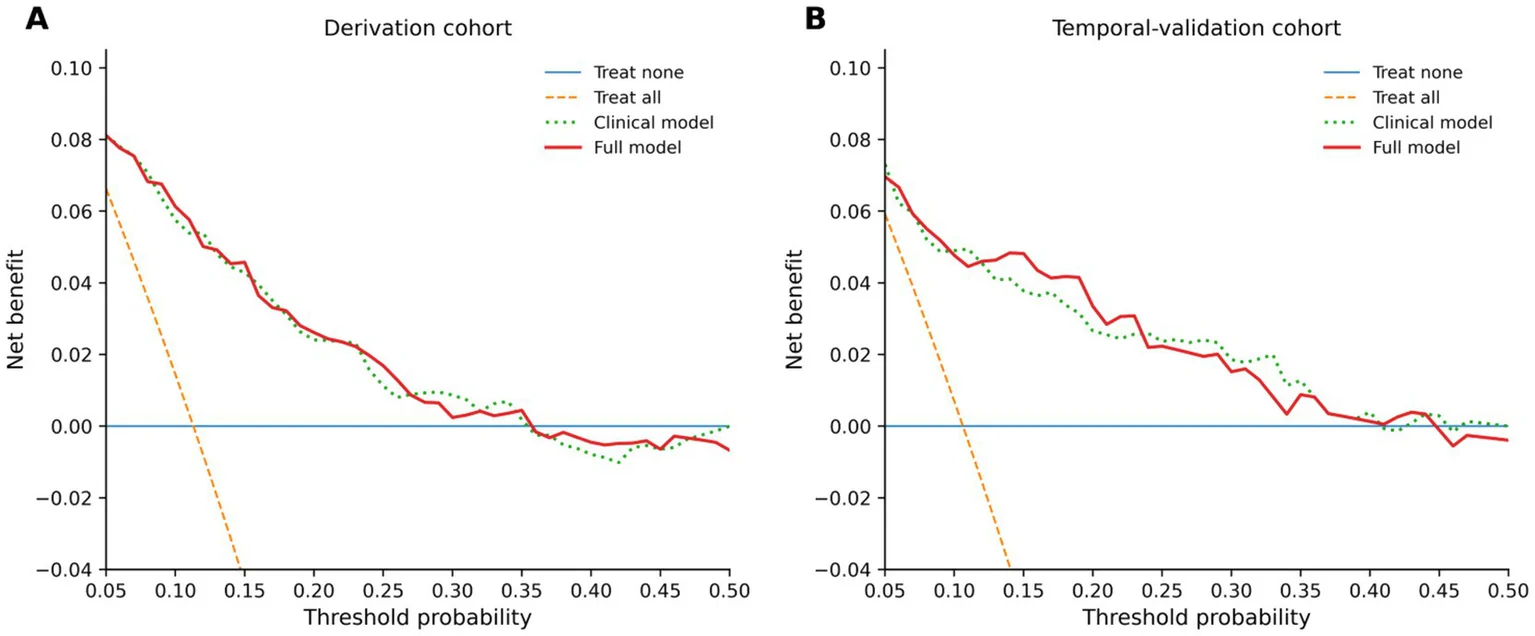
Decision-curve analysis in the derivation **(A)** and internal temporal-validation **(B)** cohorts. Curves compare the clinical model, full model, treat-all strategy, and treat-none strategy across threshold probabilities of approximately 5%–50%.

### 3.7 Complementary machine-learning analysis

An exploratory complementary gradient-boosting analysis with SHAP interpretation was conducted in the same 847-admission cohort using baseline NIHSS, AF, LVO, ln(NLR), AIP, and residual cholesterol (32). Baseline NIHSS remained the dominant feature, followed by AF and LVO, whereas ln(NLR), AIP, and residual cholesterol made smaller contributions. A concise mean-|SHAP| summary is shown in Figure 2D, while the detailed SHAP beeswarm and global-importance plot are provided in Supplementary Figure S2. This same-cohort analysis was not an independent validation and did not contribute to predictor selection, model-superiority claims, or primary inference.

### 3.8 Incremental value and sensitivity analyses

The primary incremental-value comparisons and their sensitivity analyses are summarized in Figures 2B,C. Biomarker gains were unstable across clinically relevant and data-quality sensitivity analyses. After excluding bridging thrombectomy, the derivation cohort contained 542 admissions with 47 HT events, and validation contained 229 admissions with 22 events. Validation AUC was 0.770 for the clinical model and 0.761 for the full model (ΔAUC −0.009; *p* = 0.662). In the LVO complete-case analysis, the derivation cohort included 505 admissions with 58 HT events, and the temporal-validation cohort included 220 admissions with 25 HT events. The corresponding validation AUCs were 0.800 for the clinical model and 0.815 for the full model (ΔAUC +0.015; *p* = 0.217). Restricting HT to events within 7 days yielded AUCs of 0.802 and 0.809 (ΔAUC +0.008; *p* = 0.657). Excluding three HT events lacking re-verifiable archived documentation yielded AUCs of 0.798 and 0.813 (ΔAUC +0.016; *p* = 0.479).

Under the documentation-conservative laboratory-timing approach, validation AUCs were 0.802 for the clinical model and 0.798 after adding ln(NLR). Corresponding values were 0.824 after adding AIP and residual cholesterol and 0.821 for the full model. No sensitivity analysis established a reproducible biomarker advantage. Supplementary Figure S3 provides a secondary biomarker-focused comparison of adjusted associations and nested-model AUCs using the exploratory six-variable specification.

### 3.9 Symptomatic intracranial hemorrhage: exploratory secondary analysis

The strict 7-day sICH endpoint occurred during 37 admissions, including 25 in derivation and 12 in temporal validation. Because the number of sICH events was limited, the exploratory clinical reference model was intentionally restricted to baseline NIHSS, AF, and LVO to reduce instability and obvious overfitting. This three-variable model yielded AUCs of 0.800 and 0.816, respectively. Adding ln(NLR) increased the validation AUC to 0.888 (95% CI 0.824–0.946). The increase of +0.071 was not statistically significant (DeLong *p* = 0.097). In the NLR-augmented model, baseline NIHSS was associated with sICH (OR 1.07, 95% CI 1.02–1.13; *p* = 0.010). ln(NLR) was also associated with sICH (OR 1.89, 1.04–3.42; *p* = 0.037). AF was borderline (OR 2.35; *p* = 0.055), whereas LVO was not significant. Given only 12 validation events, this finding remains exploratory and hypothesis-generating.

Batch-level distributions provided additional context for temporal drift. Median residual cholesterol was 0.325 mmol/L in the initial 431-case extraction batch and 0.825, 0.735, 0.770, and 0.730 mmol/L in four later batches. NLR missingness was 0.5% in the initial extraction batch and 29.9%–38.3% in later batches. The source-batch pattern suggests temporal differences in data capture or extraction, although the respective contributions of pre-analytical, clinical, and documentation-related factors could not be fully disentangled.

## 4 Discussion

The main finding of this study is that routine inflammatory and metabolic biomarkers did not provide statistically robust incremental predictive value beyond a clinically anchored model for in-hospital hemorrhagic transformation after intravenous thrombolysis. The six-variable clinical model retained an AUC of 0.801 in temporal validation, whereas the full model increased the AUC by 0.018, a difference that was not statistically significant. Calibration and decision-curve performance were also similar, and the small changes in discrimination were not reproduced consistently across sensitivity analyses. Baseline NIHSS and atrial fibrillation were the clearest adjusted clinical signals, a pattern also visible in the integrated summary in Figure 2. The exploratory SHAP analysis provided a concordant feature-contribution view, with NIHSS ranking highest, but it used the same cohort and a different six-variable specification and therefore does not constitute independent validation. Taken together, these results do not imply that the biomarkers are biologically irrelevant; rather, they show that association with hemorrhagic transformation does not necessarily translate into meaningful incremental prediction beyond readily available clinical information.

The inflammatory findings illustrate this distinction particularly well. Previous cohort studies and meta-analytic evidence have linked higher NLR with hemorrhagic transformation after intravenous thrombolysis, and a recent externally validated study again identified NLR among predictors of post-IVT HT (10, 34, 35). The broader prognostic relevance of admission inflammatory indices is also supported by the CLOT-VENUS substudy and by meta-analytic data for SII and SIRI, although these studies addressed different cerebrovascular populations and outcomes (25, 36). In the present cohort, ln(NLR) was not independently associated with the broader in-hospital HT endpoint and increased temporal-validation AUC by only 0.017. By contrast, the signal was stronger for strict 7-day sICH: ln(NLR) remained associated after adjustment, with an OR of 1.89, and the validation AUC increased from 0.816 to 0.888. This pattern is biologically plausible because neutrophil activation, matrix metalloproteinases, and related inflammatory pathways may promote blood–brain barrier injury (9, 37). Nevertheless, only 12 sICH events occurred in temporal validation and the AUC increment did not reach statistical significance. The NLR-sICH result should therefore be regarded as an endpoint-specific hypothesis for future study rather than evidence of clinical readiness.

The lipid-related findings require similar restraint. Lower cholesterol has previously been associated with hemorrhage after thrombolysis and with hemorrhagic transformation in selected stroke subtypes, while more recent studies have reported inverse associations for AIP and remnant cholesterol in acute ischemic stroke (11–14). Our data reproduced the direction of some of these unadjusted lipid signals, but neither AIP nor residual cholesterol remained independently associated with HT in the full model, and adding the two markers to the clinical model changed the temporal-validation AUC by only 0.002. This weak incremental performance was not readily explained by mathematical redundancy: LDL-C was measured directly, the correlation between AIP and residual cholesterol was moderate, and all variance-inflation factors were below 1.6. Temporal instability was more notable. Median residual cholesterol rose from 0.39 mmol/L in derivation to approximately 0.78 mmol/L in validation, and similar shifts were seen across extraction batches despite an unchanged LDL-C assay method. Acute-phase changes, fasting status, nutritional state, stroke phenotype, and differences in data capture may all contribute. These observations make causal interpretation of the inverse lipid pattern uncertain and emphasize that an apparent biomarker association should be tested for out-of-time incremental value before it is incorporated into a more complex prediction model.

The chronological split also provided information beyond a conventional random train-test division. The study period encompassed an institutional EMR migration in July 2024. Changes in data capture and extraction across platforms may have contributed to temporal differences in variable completeness. The laboratory analyzers used for the hematological and lipid measurements remained unchanged across the migration. The observed biomarker distribution shifts therefore cannot be readily attributed to changes in laboratory instrumentation alone and may reflect a combination of clinical, pre-analytical, and data-capture factors. Derivation-fitted multiple imputation reduces information leakage but cannot correct systematic changes in what is measured, ordered, or retained in the source system (27). Against this changing background, the clinical model remained reasonably stable while biomarker gains did not, suggesting that routinely collected clinical features were more stable over time within this institution. This is internal temporal validation rather than geographic external validation (15, 37), but it is clinically relevant because laboratory availability and workflow often change over calendar time. Existing bleeding scores such as HAT and SEDAN, and the broader SPAN-100 prognostic index, also illustrate how much risk information is already captured by clinical severity, glucose, age, and imaging features (7, 38). Recent systematic reviews of HT prediction models and risk factors have highlighted substantial heterogeneity, frequent risk of bias, limited external validation, and the continuing importance of established clinical predictors (39–41). The present findings therefore support a simple principle for future biomarker studies: new markers should demonstrate reproducible gain over a credible clinical baseline, rather than being judged primarily by univariable association or an isolated AUC. Promising signals from specialized proteins such as GFAP and UCH-L1 and from routine composites such as the fibrinogen-to-albumin ratio should be evaluated in the same way (42, 43). None of the models examined here was designed as a treatment rule, and these results should not be used to withhold intravenous thrombolysis from otherwise eligible patients.

Several features strengthen the interpretation of these findings. The analysis was designed around incremental predictive value rather than biomarker association alone, used a strictly chronological derivation-validation split after source reconciliation, and estimated preprocessing and imputation parameters in the derivation cohort before applying them to the later cohort. Model assessment extended beyond discrimination to calibration, Brier score, and decision curves, while a full-pipeline bootstrap analysis provided an additional check on overfitting. The consistency of the overall conclusion across exclusions for bridging thrombectomy, complete-case LVO analysis, a 7-day HT window, documentation-conservative laboratory timing, and outcome-verification sensitivity analyses further reduces the likelihood that the main result reflects a single analytical choice. These features are particularly important for a limited incremental-value finding because they help distinguish a genuinely small biomarker contribution from a conclusion driven by one model specification or one validation metric.

Several limitations remain. The retrospective single-center design limits generalizability and is susceptible to selection bias and institution-specific practice effects. HT ascertainment relied on official radiology reports; archived images were unavailable for blinded dual review or inter-rater assessment. Clinically triggered repeat imaging may have caused differential ascertainment and strengthened the NIHSS association. Because targeted source-record re-abstraction was not blinded to outcome status, differential information bias cannot be excluded. The primary endpoint was in-hospital HT rather than a uniform early-window endpoint, although restriction to HT documented within 7 days did not materially change the result. Although the analyzed hematological and lipid variables were derived from initial emergency specimens collected before IVT, exact draw-to-bolus intervals were not retained for every admission, and not all laboratory results were necessarily available before treatment. The findings should therefore not be interpreted as validation of a real-time pre-bolus decision tool. Individual alteplase-versus-tenecteplase identity could not be reconstructed reliably for every historical admission and was therefore not modeled. Predictor missingness and biomarker distributions also varied over time in ways that multiple imputation cannot fully resolve. In addition, 67 HT events were available in derivation for the nine-parameter full model; the post-hoc Riley assessment and bootstrap results therefore indicate limited coefficient precision and some risk of overfitting. The exploratory sICH analysis was especially event-limited, with only 12 events in temporal validation. Finally, advanced imaging markers and long-term functional outcomes were not available, and internal temporal validation cannot establish transportability across institutions. Larger multicenter cohorts with standardized predictor sampling relative to treatment and outcome ascertainment, together with external validation, are needed to determine whether more stable biomarker panels can add clinically useful information beyond established clinical predictors.

## 5 Conclusion

In this single-center cohort with internal temporal validation, clinical severity and established clinical features accounted for most of the usable predictive information for in-hospital hemorrhagic transformation after intravenous thrombolysis. Adding ln(NLR), AIP, and residual cholesterol produced only small, non-significant gains that were not consistent across sensitivity analyses, while the NLR–sICH association remained exploratory. These findings support evaluating candidate biomarkers against strong clinical baselines and warrant confirmation in multicenter cohorts with standardized predictor sampling relative to treatment and outcome ascertainment, together with external validation.

## Data Availability

The datasets analyzed in this study are not publicly available due to privacy and institutional restrictions related to clinical patient data. Requests to access the datasets should be directed to the corresponding author, Zenghui Yue (yue5381316@126.com), and will be considered upon reasonable request subject to applicable institutional and ethical requirements.
