# Supplementary material for: Clinical severity predominates over routine inflammatory and metabolic biomarkers in predicting hemorrhagic transformation after intravenous thrombolysis: internal temporal validation of incremental biomarker value
Source: Front Neurol. 2026 Oct 21;17:1940110. doi: 10.3389/fneur.2026.1940110 (PMC13731395; doi:10.3389/fneur.2026.1940110)
Supplement: Supplementary file 1 [file Supplementary_file_1.docx]

**Supplementary Material**

*Supplementary tables:* ***3;*** *Supplementary figures:* ***3***

**Supplementary Tables**

Supplementary Table S1. Uniformly shrunken coefficients for the full nine-variable model.

| **Predictor** | **Unshrunken pooled β** | **Shrunken β** | **Shrunken OR** |
| --- | --- | --- | --- |
| Age (per +1 year) | -0.007 | -0.006 | 0.994 |
| Baseline NIHSS (per +1 point) | 0.081 | 0.070 | 1.072 |
| Atrial fibrillation (yes vs no) | 0.979 | 0.845 | 2.328 |
| Large-vessel occlusion (yes vs no) | 0.558 | 0.482 | 1.619 |
| Rapid glucose (per +1 mmol/L) | 0.000 | 0.000 | 1.000 |
| Onset-to-treatment time (per +1 h) | 0.037 | 0.032 | 1.033 |
| ln(NLR) (per +1) | 0.236 | 0.204 | 1.226 |
| AIP (per +1) | -0.351 | -0.303 | 0.738 |
| Residual cholesterol (per +1 mmol/L) | -0.122 | -0.106 | 0.900 |

The uniform shrinkage factor was 0.863, derived from the mean calibration slope across 500 bootstrap resamples. Each resample re-estimated winsorization limits, refitted iterative imputation, re-dichotomized AF and LVO, and refitted the logistic model. For each resample, performance was calculated both in the bootstrap sample and after applying the bootstrap-fitted preprocessing/model pipeline to the original derivation cohort; optimism was defined as the difference between these two estimates and averaged across resamples. The primary analysis retained 20 posterior-sampling imputations; bootstrap validation served as a supplementary overfitting and shrinkage assessment. The intercept is omitted because no deployable equation or nomogram is proposed. Unshrunken pooled coefficients are from the derivation-cohort full model in Table 2; shrunken ORs equal exp(shrunken β).

**Supplementary Table S2. Admission-date reconciliation and temporal-split audit. Cohort membership was generated from verified admission dates after deduplication.**

| **Audit item** | **Dataset result** |
| --- | --- |
| Analytical records before deduplication | 851 |
| Duplicate same-admission records removed | 4 |
| Retained unique admissions | 847 |
| Retained admissions with verified admission date | 847/847 (100%) |
| Cohort assignment method | Chronological rank by admission date |
| Chronological rank 593 | 25 Nov 2024; Derivation |
| Chronological rank 594 | 28 Nov 2024; Validation |
| Temporal split / HT events | 593 / 254; HT 67 / 27 |

**Supplementary Table S3. Selected characteristics of records with admission dates missing in the initial extraction with the remaining records in the 851-record source extract.**

| **Characteristic** | Admission dates missing in initial extraction (**n = 168**) | Admission dates available in initial extraction (**n = 683**) |
| --- | --- | --- |
| Residual cholesterol, mmol/L, median | 0.77 | 0.46 |
| NLR, median | 3.05 | 2.62 |
| LDL-C, mmol/L, median | 2.59 | 2.73 |
| Hypertension, % | 71.8 | 80.6 |
| Hemorrhagic transformation, % | 10.2 | 11.3 |

This descriptive source-data audit compares the 168 records with missing admission dates in the initial extraction against the other 683 records before source-date verification and deduplication. It identified differences in selected data fields, particularly residual cholesterol and hypertension, whereas HT proportions were similar (10.2% versus 11.3%). Missing dates reflected incomplete analytical extraction rather than absent hospital admission dates. Because this comparison used the original 851-record extract, it was not treated as a validation-cohort analysis; the primary analysis used 847 dated, deduplicated admissions.

**Supplementary Figures**

**Supplementary Figure S1. Decision-curve analysis of the exploratory six-variable biomarker-focused model in the 847-admission cohort. (A)** Derivation cohort (n = 593; 67 HT events). **(B)** Internal temporal-validation cohort (n = 254; 27 HT events). In this secondary specification, the three-variable clinical reference included baseline NIHSS, AF, and LVO, and the six-variable biomarker-focused model additionally included ln(NLR), AIP, and residual cholesterol. In the figure legend, “Clinical model” denotes this three-variable clinical reference and “Combined model” denotes the six-variable biomarker-focused model. This secondary specification is not the primary six-variable clinical-anchor model used in the main analysis. Curves show these two models with treat-all and treat-none strategies.

**Supplementary Figure S2.** **Detailed exploratory complementary SHAP interpretation of the six-variable biomarker-focused gradient-boosting model in the same 847-admission cohort.** The six variables were baseline NIHSS, AF, LVO, ln(NLR), AIP, and residual cholesterol. **(A)** SHAP beeswarm; the horizontal axis shows contributions to the log-odds of HT, and color indicates feature value. **(B)** Feature importance ranked by mean absolute SHAP value. Figure 5D provides a concise main-text summary of the mean absolute SHAP values. This same-cohort analysis is not an independent validation of the primary nested logistic models and did not inform predictor selection or model-superiority claims.

**Supplementary Figure S3. Secondary exploratory summary of the six-variable biomarker-focused specification in the 847-admission cohort. (A)** Adjusted odds ratios with 95% confidence intervals for ln(NLR), AIP, residual cholesterol, baseline NIHSS, LVO, and AF. **(B)** AUCs for the three-variable clinical reference, inflammation-augmented, metabolic-augmented, and six-variable combined models in the derivation and internal temporal-validation cohorts. In the panel labels, “Clinical” denotes the three-variable clinical reference and “Combined” denotes the six-variable biomarker-focused model. This secondary analysis is provided only as a robustness comparison and is not the primary clinically anchored framework.
